# Assessment of the *Artemia salina* toxicity assay as a substitute of the mouse lethality assay in the determination of venom-induced toxicity and preclinical efficacy of antivenom

**DOI:** 10.1016/j.toxcx.2024.100195

**Published:** 2024-04-03

**Authors:** Xavier Araya, Mitchel Okumu, Gina Durán, Aarón Gómez, José María Gutiérrez, Guillermo León

**Affiliations:** a Instituto Clodomiro Picado, Facultad de Microbiología, Universidad de Costa Rica, San José, Costa Rica; b Department of Public Health, Pharmacology and Toxicology, University of Nairobi, Kenya

**Keywords:** Antivenom, *Artemia salina* toxicity test, Neutralization of lethality, Preclinical efficacy, Snake venom

## Abstract

Mice are routinely used in snake venom research but are costly and subject to pain and suffering. The crustacean *Artemia salina* could be an alternative to mice, but data to support its adoption in snake venom research is limited. The aim of the present study was to evaluate the suitability of *A. salina* as a surrogate of mice in assessing the toxicity of venoms and the preclinical efficacy of antivenoms. The toxicity of venoms from 22 snakes of medical importance in sub–Saharan Africa was evaluated in mice (intraperitoneally; i.p. and intravenously; i.v.) and in *A. salina*. Subsequently, the capacity of a commercial antivenom to neutralize the toxicity of these venoms in mice and *A. salina* was investigated. There was a positive correlation between the i.v. median lethal doses (LD_50s_) and the i.p. LD_50s_ in mice (r = 0.804; p < 0.0001)*,* a moderate correlation between the i.v. LD_50s_ in mice and the median lethal concentrations (LC_50s_) in *A. salina* (r = 0.606; p = 0.003)*,* and a moderate correlation between the i.p. LD_50s_ in mice and the LC_50s_ in *A. salina (*r = 0.426; p = 0.048). Moreover, there was a strong correlation between the i.p. median effective doses (ED_50s_) and the i.v. ED_50s_ in mice (r = 0.941, p < 0.0001)*,* between the i.p. ED_50s_ in mice and the ED_50s_ in *A. salina* (r = 0.818, p < 0.0001)*,* and between the i.v. ED_50s_ in mice and the ED_50s_ in *A. salina* (r = 0.972, p < 0.0001). These findings present *A. salina* as a promising candidate for reducing reliance on mice in snake venom research. Future investigations should build upon these findings, addressing potential limitations and expanding the scope of *A. salina* in venom research and antivenom development.

## Highlights

• The correlation between the mouse lethality and the *Artemia salina* toxicity tests was assessed using African snake venoms.
• A significant correlation was observed between LD_50_s when using the intravenous and intraperitoneal routes in mice.
• A significant correlation was observed when comparing LD_50_s in mice and LC_50_s in *A. salina* model.
• A highly significant correlation was observed when comparing antivenom efficacy (ED_50_) in these two models.

## Graphical abstract

**Figure:**
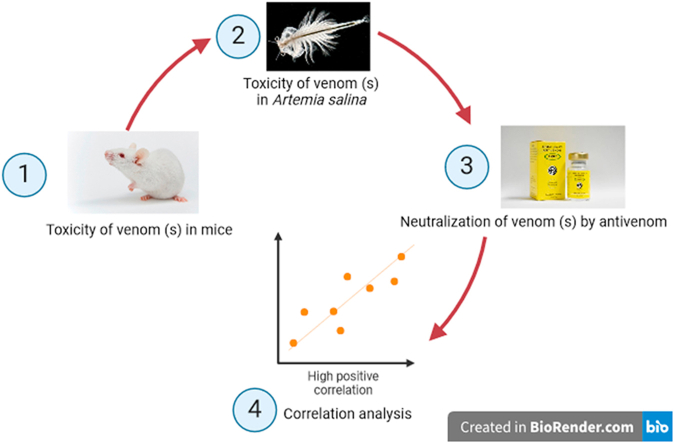

## 1 Introduction

The biomedical sciences, including anatomy and physiology, disease pathogenesis, surgical technique, and pharmaceutical development, have all benefited greatly from modelling in experimental animals (Bernard, 1957; Robinson et al., 2019). However, the pain, anxiety, distress, and long-term suffering that experimental animals endure creates an ethical dilemma between the use of these animals to further biological research and the welfare of the research subjects (Robinson et al., 2019).

The use of the 3Rs principle—replacement, reduction, and refinement—may result in more humane animal research (Russell and Burch, 1959). Nonetheless, there are certain areas of study where applying this idea has been challenging. For instance, mouse models are the main tool used in snake venom research to assess venom toxicity and the preclinical efficacy of antivenoms (World Health Organization, 2017). Since mice are used frequently in snake venom research, the WHO Guidelines for the Production, Control, and Regulation of Snake Venom Immunoglobulins heavily reference the model despite its limitations in antivenom efficacy evaluation (World Health Organization, 2017; Gutiérrez et al., 2021; Silva et al., 2022).

Mice are injected with different doses of venom in the mouse lethality assay, and they are then monitored for 24–48 h (World Health Organization, 2017). The mortality data obtained from these observations is used to determine the median lethal dose, or LD_50_—the amount of venom that causes 50% of the injected animals to die (World Health Organization, 2017). On the other hand, mice are injected with an incubated mixture of a constant challenge dose of venom (3–6 LD_50_s) and graded dilutions of antivenom to study the neutralizing capacity of antivenoms (World Health Organization, 2017). The effectiveness of the antivenom in mitigating venom-induced lethality is then assessed over 24- to 48-h and expressed as the median effective dose (ED_50_), which is the volume of antivenom or venom/antivenom ratio at which 50% of challenged animals survive (World Health Organization, 2017).

A number of improvements have been made to the mouse lethality test, such as the use of analgesics (Chacón et al., 2015; Herrera et al., 2018), reducing the time of the assay (Barber et al., 2014; Durán et al., 2021), and lowering the number of animals needed to produce reliable results (Solano et al., 2010). Additionally, some authors have presented alternative models, including the use of embryonated eggs (Verity et al., 2021), cell-based assays (Lopes-de-Souza et al., 2019), antivenomics (Gutiérrez et al., 2014; Pla et al., 2017), and *in vitro* methods such as the indirect hemolytic activity assay (Habermann and Hardt, 1972; Gutiérrez et al., 1988; Barbosa et al., 1995) and the enzyme linked immunosorbent assay (Heneine et al., 1998; Liu et al., 2021). Despite their limitations, these studies have demonstrated noteworthy correlations with the mouse lethality assay for specific venom-antivenom combinations.

Okumu and co-workers have recently used the *Artemia salina* animal model to determine the neutralization capacity of two antivenoms (Okumu et al., 2020). A follow-up study showed that the *A. salina* model was better at predicting venom-induced dermonecrosis than lethality in mice (Okumu et al., 2021). This test involves exposing hatched larvae of *A. salina* to graded doses of venom over 24 h and observing the number of dead larvae (Okumu et al., 2020). The mortality data is used to calculate the median lethal concentration (LC_50_), i.e., the concentration of venom that causes the death of 50% of *A. salina* larvae. To determine the neutralizing efficacy of antivenom, the larvae are exposed to a constant challenge concentration of venom mixed with different dilutions of antivenom (Okumu et al., 2020). The number of dead larvae after 24 h is used to calculate the ED_50_.

The *A. salina* toxicity test has several advantages over the mouse assay, including ease of use, low cost, quick findings, and the ability to examine a large number of samples, in addition to ethical benefits (Freires et al., 2016). It was first presented by Meyer and colleagues (Meyer et al., 1982), and since then it has been extensively used in toxicology (Kerster and Schaeffer, 1983; Sanchez-Fortun and Barahona, 2009; Hamidi et al., 2014; Freires et al., 2023). However, its application in snake venom research is limited (Okumu et al., 2020, 2021). The present study sought to investigate the suitability of *A. salina* as a surrogate model for mice in evaluating the toxicity of snake venom and the preclinical efficacy of antivenom.

## 2 Materials and methods

### 2.1 Ethics

This study was approved by the Institutional Committee for the Care and Use of Laboratory Animals (CICUA) of Universidad de Costa Rica (reference numbers 82-08 and 39-20) and met the International Guiding Principles for Biomedical Research Involving Animals (Bankowski and Howard-Jones, 1985).

### 2.2 Venom

The batch numbers and geographical origins of venoms used in this study are summarized in Table 1. The collected venoms were lyophilized and stored at −40 °C. Lyophilized venom was weighed and dissolved in 0.12 M NaCl, 0.04 M phosphate buffer, pH 7.2 (PBS) at the time of use.

**Table 1:** Details of the venoms used in toxicity evaluation and preclinical antivenom efficacy assessment.

Genera	Species	Batch number^a^	Geographic origin
*Bitis*	*B. arietans*	322.061	Unspecified
	*B. gabonica*	725.031	Unspecified
	*B. nasicornis*	500.102	Unspecified
	*B. rhinoceros*	701.070	Ghana
*Echis*	*E. leucogaster*	623.070	Mali
	*E. ocellatus*	216.031	Unspecified
	*E. pyramidum*	523.070	Egypt
*Naja*	*N. ashei*	410.191	Kenya
	*N. katiensis*	705.010	Burkina Faso
	*N. mossambica*	627.002	Tanzania
	*N. nigricinta*	507.081	South Africa
	*N. nigricollis*	616.031	Unspecified
	*N. anchietae*	527.002	Namibia
	*N. annulifera*	622.040	Mozambique
	*N. haje*	222.061	Unspecified
	*N. melanoleuca*	516.031	Unspecified
	*N. nivea*	524.010	South Africa
	*N. senegalensis*	805.101	Mali
*Dendroaspis*	*D. angusticeps*	305.000	Tanzania/Mozambique
	*D. jamesonii*	923.011	Cameroon
	*D. polylepis*	416.031	Unspecified
	*D. viridis*	516.001	Ghana

a All venoms were obtained from Latoxan (Portes-dès Valence, France) (https://www.latoxan.com/).

### 2.3 Snake antivenom

EchiTAb-plus-ICP antivenom (batch 6640421PALQ, which has an expiration date of April 2024 and protein content of 7.3 ± 0.2 g/dL, and batch 6771021PALQ, which has an expiration date of October 2024 and protein content of 7.2 ± 0.1 g/dL) were used in this study. This antivenom is a polyspecific formulation of whole immunoglobulin G (IgG) from the plasma of horses immunized with venoms of *B. arietans*, *E. ocellatus*, *N. nigricollis*, and *Dendroaspis polylepis,* and purified by caprylic acid precipitation (Rojas et al., 1994). It is effective in neutralizing the venoms of several species of *Echis* spp, *Bitis* spp, *Naja* spp and *D. polylepis* (Gutiérrez et al., 2005; Segura et al., 2010; Petras et al., 2011).

### 2.4 Determination of the LD_50_ of the snake venoms in mice

Groups of eight CD-1 mice of both sexes were pretreated with a 50 mg/kg subcutaneous dose of tramadol (Chacón et al., 2015). After 15 min, the mice received different amounts of venom dissolved in PBS via the intravenous (i.v.) or intraperitoneal (i.p.) route. The weight range of mice that received venom intraperitoneally was between 16 and 18 g, while the weight range of mice that received venom intravenously was between 20 and 22 g. The volume of injection was 0.2 mL for the i.v. route and 0.5 mL for the i.p. route. The number of deaths after 24 h (i.v.) or 48 h (i.p.) was recorded. The LD_50_ and the corresponding 95% Confidence Intervals (95% CI) were calculated using Probit Regression Analysis (Finney, 1971) and expressed as milligrams of venom per kilogram body weight of mouse (mg venom/kg bwt) that killed 50% of the injected mice.

### 2.5 Determination of the LC_50_ of the snake venoms in *A. salina*

The method of Meyer and colleagues was used with slight modifications (Meyer et al., 1982). Briefly, 1.5 mL of PBS containing different amounts of venoms were mixed with ten 48-hr old *A. salina* larvae suspended in 0.5 mL of sterile sea water having a NaCl concentration of 0.42 M. These mixtures were incubated at room temperature and the larvae were observed after 24 h. The number of dead larvae, i.e., larvae that did not move during 2 min, was recorded and used to calculate the LC_50_ by Probit regression analysis (Finney, 1971).

### 2.6 Determination of the capacity of antivenom to neutralize venom-induced lethality in mice

Aliquots containing a constant challenge dose of venom and variable dilutions of antivenom were incubated at 37 °C for 30 min and injected in mice i.p. or i.v. The challenge dose was 3LD_50_s for venoms of *Naja* spp and *Dendroaspis* spp or 5LD_50_s for venoms of *Bitis* spp and *Echis* spp (Gutiérrez et al., 2005; Segura et al., 2010). Control group mice received venom only dissolved in PBS. The volume of injection was 0.2 mL for the i.v. route and 0.5 mL for the i.p. route. The number of deaths were recorded after 24 h (when the i.v. route was used) or 48 h (when the i.p. route was used). The ED_50_, expressed as mg venom/mL of antivenom, and the corresponding 95% confidence intervals (CI), were calculated by Probit Regression Analysis (Finney, 1971).

### 2.7 Determination of the capacity of the antivenom to neutralize venom-induced lethality in *A. salina*

The method described by Okumu et al. was used (Okumu et al., 2020), with modifications. Briefly, 0.5 mL of PBS, containing a constant challenge dose of venoms (2–6 LC_50_s depending on the venom), were mixed with 1.0 mL of different dilutions of the antivenom. Antivenom was previously dialyzed using a dialysis tubing cellulose membrane (D9527; Sigma Aldrich, St Louis, MO, USA) in a volume of distilled water corresponding to 40 times the volume of antivenom (for the first two cycles), and 0.15 M NaCl (for the last cycle) to remove phenol (a preservative), as it is toxic to the *A. salina* larvae. The antivenom was then concentrated by freeze-drying and dissolved in distilled water to attain the same protein concentration as the original antivenom. Venom-antivenom mixtures were prepared and incubated at room temperature for 30 min. Then, mixtures were centrifuged at 17,700×*g* for 6 min to avoid interference in the assay due to turbidity and immune complex precipitation. Ten *A. salina* larvae were suspended in 0.5 mL of sterile sea water and added to the venom-antivenom mixtures (1.5 mL), followed by incubation for 24 h at room temperature. *A. salina* larvae exposed to venom only served as control. Mortality was recorded after 24 h and the ED_50_ and 95% CI were calculated by probit regression analysis. ED_50_ corresponds to the venom/antivenom ratio at which 50% of the *A. salina* larvae survived.

### 2.8 Statistical analysis

The LD_50_ and LC_50_ of the venoms and the ED_50_ of the antivenom (and their corresponding 95% confidence intervals) were determined by Probit Regression Analysis. Pearson's bivariate correlations evaluated the relationship between lethality in mice (i.v./i.p. LD_50_) and lethality in *A. salina* (LC_50_) as well as the relationship between the ED_50_ of antivenom determined from mice and *A*. *salina*. In the case of venoms which were not neutralized by the antivenom, i.e., the values of ED_50_s could not be calculated, these data were not used for the correlation analysis. Data analysis was carried out using the Statistical Package for the Social Sciences (IBM, Version 25).

## 3 Results

### 3.1 Toxicity of the venoms in mice and in *A. salina*

Table 2 shows the results for the i.v. LD_50_, i.p. LD_50_, and LC_50_ of the 22 studied venoms. According to the i.v. LD_50_ data, *D. polylepis* venom was the most toxic with an LD_50_ of 0.31 mg/kg (0.29–0.35) while *N. annulifera* venom was the least toxic with an LD_50_ of 3.47 mg/kg (2.76–5.44). According to the i.p. LD_50_ data, *D. polylepis* venom was the most toxic with an LD_50_ of 0.26 mg/kg (0.18–0.34) while *N. anchietae* was the least toxic with an LD_50_ of 3.85 mg/kg (2.84–4.99) (Table 2). The i.p. LD_50_/i.v. LD_50_ ratio for viperids ranged from 1.41 *(B. gabonica)* to 3.70 *(E. pyramidum)* while in elapids it ranged from 0.80 *(N. haje)* to 1.74 *(N. nigricinta)*. Moreover, according to LC_50_ data from the *A. salina* model, *D. angusticeps, D. jamesoni, D. viridis,* and *N. nigricollis* venoms were the most toxic and had similar LC_50_s, i.e. 0.01 (0.00–0.01) mg/mL for *D. angusticeps*, 0.01 (0.00–0.02) mg/mL for *D. jamesonii*, 0.01 (0.00–0.02) mg/mL for *D. viridis*, and 0.01 (0.00–0.02) mg/mL for *N. nigricollis*, while *N. annulifera* venom was the least toxic venom with an LC_50_ of 0.24 (0.16–0.34) mg/mL (Table 2).

**Table 2:** A comparison of the toxicity (lethality) of venom from 22 snakes of medical importance in sub-Saharan Africa.

Genera	Species	Mouse			*A. salina*
		i.v. LD_50_ (mg/kg)	i.p. LD_50_ (mg/kg)	i.p. LD_50_/iv. LD_50_	LC_50_ (mg/mL)
*Bitis*	*B. arietans*	0.54 (0.40–0.75)	1.23 (0.98–1.49)	2.28	0.05 (0.02–0.07)
	*B. gabonica*	0.98 (0.86–1.12)	1.38 (0.93–1.85)	1.41	0.07 (0.04–0.10)
	*B. nasicornis*	0.92 (0.82–1.09)	1.48 (0.86–1.95)	1.61	0.09 (0.06–0.16)
	*B. rhinoceros*	0.85 (0.72–0.97)	1.42 (0.94–2.08)	1.67	0.05 (0.02–0.09)
*Echis*	*E. leucogaster*	1.44 (1.04–1.96)	2.31 (1.55–3.07)	1.60	0.09 (0.04–0.13)
	*E. ocellatus*	0.87 (0.81–0.94)	1.84 (1.24–2.89)	2.11	0.05 (0.04–0.07)
	*E. pyramidum*	0.61 (0.45–0.83)	2.26 (1.51–3.84)	3.70	0.05 (0.04–0.07)
*Naja*	*N. ashei*	0.88 (0.61–1.24)	1.26 (0.76–1.98)	1.43	0.02 (0.01–0.04)
	*N. katiensis*	0.88 (0.65–1.18)	1.23 (0.86–1.79)	1.40	0.02 (0.01–0.03)
	*N. mossambica*	0.99 (0.87–1.14)	1.08 (0.81–1.45)	1.09	0.02 (0.01–0.04)
	*N. nigricinta*	0.77 (0.67–0.88)	1.34 (0.86–2.24)	1.74	0.04 (0.02–0.06)
	*N. nigricollis*	0.94 (0.84–1.07)	1.08 (0.63–1.55)	1.15	0.01 (0.00–0.02)
	*N. anchietae*	2.46 (1.66–3.72)	3.85 (2.84–4.99)	1.57	0.04 (0.02–0.06)
	*N. annulifera*	3.47 (2.76–5.44)	3.03 (1.77–4.34)	0.87	0.24 (0.16–0.34)
	*N. haje*	0.56 (0.44–0.85)	0.45 (0.35–0.49)	0.80	0.05 (0.03–0.08)
	*N. melanoleuca*	0.33 (0.25–0.44)	0.48 (0.38–0.69)	1.45	0.07 (0.05–0.10)
	*N. nivea*	1.59 (1.37–1.84)	1.32 (1.05–1.66)	0.83	0.02 (0.01–0.04)
	*N. senegalensis*	0.50 (0.35–0.64)	0.45 (0.35–0.61)	0.90	0.02 (0.00–0.01)
*Dendroaspis*	*D. angusticeps*	1.40 (1.23–1.60)	2.10 (1.76–2.31)	1.50	0.01 (0.00–0.01)
	*D. jamesonii*	1.01 (0.93–1.19)	1.09 (0.84–1.56)	1.08	0.01 (0.01–0.02)
	*D. polylepis*	0.31 (0.29–0.35)	0.26 (0.18–0.34)	0.84	0.02 (0.01–0.03)
	*D. viridis*	0.43 (0.32–0.74)	0.52 (0.48–0.57)	1.20	0.01 (0.00–0.02)

*Lethality was expressed as the Median Lethal Dose (LD_50_) in the mouse model (mg/kg body weight), or the median Lethal Concentration (LC_50_) (mg/mL) in the brine shrimp model. In the three cases, the 95% CIs are shown in parenthesis.

### 3.2 Relationship between the lethality of the venoms in mice and *A. salina*

**Fig. 1:**
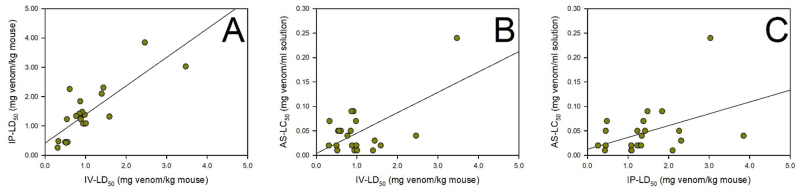
Pearson's bivariate correlations between the i.v. LD_50_ and i.p. LD_50_ of snake venoms in mice (r = 0.804, n = 22, p < 0.0001) (A), the iv LD_50_ in mice and the LC_50_ in *A. salina* (r = 0.606, n = 22, p < 0.003) (B), and the i.p. LD_50_ and the LC_50_ in *A. salina* (r = 0.426, n = 22, p < 0.048) (C). i.v.: intravenous, i.p.: intraperitoneal, LD_50_: median lethal dose, LC_50_: median lethal concentration.

There was a strong and significant correlation between the i.v. LD_50_ and the i.p. LD_50_ of the studied venoms in mice (r = 0.804, n = 22, p < 0.0001) (Fig. 1A), and a moderate and significant correlation between the i.v. LD_50_ values of the venoms in mice and the LC_50_ of the venoms in *A. salina* (r = 0.606, n = 22, p = 0.003) (Fig. 1B). There was a moderate and significant correlation between the i.p. LD_50_ values of the venoms in mice and the LC_50_ of the venoms in *A. salina* (r = 0.426, n = 22, p = 0.048) (Fig. 1C).

### 3.3 Neutralization capacity of antivenom against venom-induced lethality in mice and *A. salina*

According to neutralization data from the i.v. ED_50_ protocol, the test antivenom was most effective in neutralizing *E. ocellatus, B. arietans*, and *B. rhinoceros* venoms but did not neutralize the venoms of *D. angusticeps, D. jamesoni, N. nivea, N. anchieta, N. annulifera, B. nasicornis* and *E. leucogaster* at the lowest venom/antivenom ratios tested (Table 3). According to neutralization data from the i.p. ED_50_ protocol, the test antivenom was most effective in neutralizing *B. arietans, E. ocellatus,* and *E. pyramidum* venoms but did not neutralize the venoms of *N. haje, N. anchietae, N. annulifera, D. angusticeps,* and *D. jamesoni* at the lowest venom/antivenom ratios tested (Table 3). According to neutralization data from the ED_50_ protocol in *A. salina*, the test antivenom was the most effective in neutralizing *E. ocellatus, B. arietans,* and *B. rhinoceros* venoms but failed to neutralize *D. angusticeps* and *D. jamesoni* venoms. (Table 3).

**Table 3:** Capacity of the antivenom to neutralize venom-induced lethality in mice and *A. salina*.

Genera	Species	Animal model		
		Mouse^a^		*A. salina*^b^
		i.v. ED_50_	i.p. ED_50_	ED_50_
*Bitis*	*B. arietans*	5.00 (4.00–7.20)	5.50 (4.40–7.00)	3.30 (2.10–6.70)
	*B. gabonica*	1.20 (0.70–2.30)	3.00 (1.90–5.00)	1.50 (0.20–3.70)
	*B. nasicornis*	<0.75	3.20 (1.90–5.00)	0.75 (0.03–1.40)
	*B. rhinoceros*	3.00 (2.00–5.60)	3.40 (2.70–4.40)	3.20 (2.10–5.20)
*Echis*	*E. leucogaster*	<3.00	3.20 (1.80–5.10)	1.60 (0.80–4.50)
	*E. ocellatus*	5.10 (3.90–6.90)	4.30 (2.90–5.70)	4.10 (2.00–6.60)
	*E. pyramidum*	2.90 (2.10–3.80)	3.90 (2.50–4.90)	2.80 (1.40–5.10)
*Naja*	*N. ashei*	0.49 (0.37–0.59)	0.89 (0.60–1.31)	0.90 (0.60–1.50)
	*N. katiensis*	0.73 (0.55–0.99)	0.36 (0.25–0.53)	1.10 (0.60–2.00)
	*N. mossambica*	0.84 (0.62–1.59)	0.58 (0.33–0.87)	1.20 (0.80–1.70)
	*N. nigricinta*	0.69 (0.54–0.89)	0.67 (0.42–0.94)	1.00 (0.80–1.40)
	*N. nigricollis*	1.00 (0.70–1.50)	0.74 (0.33–1.11)	1.50 (0.90–2.00)
	*N. anchietae*	<0.60	<1.00	0.80 (0.10–2.00)
	*N. annulifera*	<1.00	<0.70	0.60 (0.10–1.20)
	*N. haje*	0.10 (0.07–0.20)	<0.08	0.90 (0.10–2.50)
	*N. melanoleuca*	0.16 (0.09–0.25)	0.17 (0.02–0.38)	1.00 (0.30–1.80)
	*N. nivea*	<1.00	0.47 (0.20–0.81)	1.20 (0.50–2.20)
	*N. senegalensis*	0.10 (0.00–0.20)	0.12 (0.06–0.18)	0.90 (0.10–2.00)
*Dendroaspis*	*D*.*angusticeps*	<0.80	<0.40	<0.19
	*D. jamesonii*	<0.30	<0.10	<0.25
	*D. polylepis*	0.10 (0.00–0.20)	0.10 (0.00–0.10)	0.50 (0.10–1.00)
	*D. viridis*	0.16 (0.11–0.21)	0.10 (0.04–0.16)	0.70 (0.40–1.30)

a mg venom/ml antivenom.

b mg venom/ml antivenom*, ED_50_: Median Effective Dose.

### 3.4 Relationship between neutralization of venom-induced lethality by antivenom in mice and *A. salina*

**Fig. 2:**
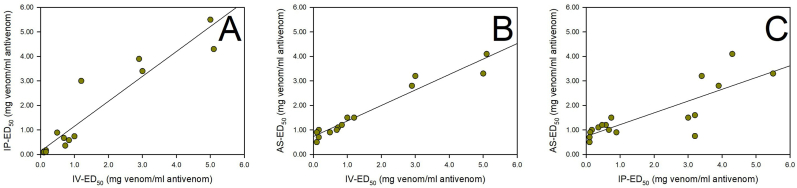
Pearson's bivariate correlations between the i.v. ED_50_ and i.p. ED_50_ in mice (r = 0.941, n = 14, p < 0.0001) (A), the i.v. ED_50_ in mice and the EC_50_ in *A. salina* (r = 0.972, n = 15, p < 0.0001) (B), and the i.p. ED_50_ in mice and the EC_50_ in *A. salina* (r = 0.818, n = 17, p < 0.0001) (C). i.v.: intravenous, i.p.: intraperitoneal, ED_50_: median effective dose.

A strong, positive, and significant correlation was observed between the i.p. ED_50_ and the i.v. ED_50_ in mice (r = 0.941, n = 14, p < 0.0001) (Fig. 2A), between the i.v. ED_50_ in mice and the ED_50_ in *A. salina* (r = 0.972, n = 15, p < 0.0001) (Fig. 2B), and between the i.p. ED_50_ in mice and the ED_50_ in *A. salina* (r = 0.818, n = 17, p < 0.0001) (Fig. 2C).

## 4 Discussion

The present study used mice and *A*. *salina* to assess the toxicity of 22 venoms from snakes of medical importance in sub–Saharan Africa. The efficacy of an antivenom routinely used in clinical practice in the region was also investigated via the two models, and the results using these models were compared to establish whether they correlated. In terms of lethality in the mouse model, our results allow the comparison between LD_50_ values by the i.v. and the i.p. routes. As a general trend, viperid venoms of the genera *Bitis* and *Echis* tend to be more toxic by the i.v. route as compared to the i.p. route, although it was observed that only in the cases of *B. arietans*, *E. ocellatus* and *E. pyramidum* was this difference significant, i.e., the 95% CI did not overlap. In the case of elapid venoms of the genera *Naja* and *Dendroaspis*, the differences between values of LD_50_ by these routes were less marked, and only in the case of *D. angusticeps* venom was there a significant difference, i.e., a lower value by the i.p. route was observed. The general trend observed towards less marked differences between lethality by these two routes of venom administration in the case of elapid venoms can be explained by the fact that lethal toxins in elapids are low molecular weight neurotoxins (6–9 kDa) known to have higher bioavailability regardless of the route of injection (Oukkache et al., 2014).

Correlations were observed between the toxicity of the venoms in mice and in *A*. *salina*. The mechanism of venom-induced lethality in *A*. *salina* is not clear. However, in the case of predominantly neurotoxic elapids, venom-induced lethality may be due to three finger neurotoxins acting at the neuromuscular junctions. On the other hand, the cytotoxic activities of snake venom metalloproteases (SVMPs), phospholipase A_2_s (PLA_2_s) and cytotoxic three finger toxins (3FTxs) in predominantly cytotoxic elapids and viperids may be responsible for venom-induced lethality probably through the damage of tissues in *A. salina*. Further work is necessary to identify the components in a variety of venoms which are responsible for toxicity in *A. salina*, in order to have an in-depth characterization of this experimental model of toxicity. This could be achieved by determining the ‘toxicity score’ of venom fractions in *A. salina* (Laustsen et al., 2015).

Viperid venom-induced lethality in mice after i.v. administration has been associated with the procoagulant effects of the venom, which causes rapid intravascular thrombosis induced by the procoagulant snake venom serine proteases (SVSPs) and metalloproteases (SVMPs) (Gutiérrez et al., 2017; Offor et al., 2022). In addition, massive systemic hemorrhage induced by SVMPs and toxins affecting hemostasis may also contribute to lethality by the i.v. route. On the other hand, viper venom-induced lethality in mice after i.p. administration is likely a consequence of massive extravasation secondary to the hemorrhagic action of SVMPs and the increase in vascular permeability induced by SVMPs, SVSPs and PLA_2_s (Chacón et al., 2015; Gutiérrez et al., 2017). However, other mechanisms might play a role in lethality, and these may vary from venom to venom. It is not clear how these different mechanisms converge resulting in the significant correlations observed in LD_50_ when the i.v. or i.p. routes are used.

The most striking finding of our study was the high correlation between mouse and *A. salina* models when the neutralizing ability of the antivenom was evaluated. It is noteworthy that the correlations between the models were higher when comparing the values of ED_50_s than when comparing the values of LD_50_s. Therefore, regardless of the mechanisms of toxicity operating in the two models, the high correlation described for the estimation of ED_50_ values of the antivenom suggests that the *A. salina* toxicity assay could be a suitable alternative to the mouse assays in some stages of the routine quality control of antivenoms, for example, in the assessment of the neutralizing ability of raw hyperimmune plasma and the verification of the specification fulfillment of bulk batches. In the context of the 3Rs principle, the *A. salina* toxicity assay may represent a positive step forward in the road to replace the mouse lethality assays in the assessment of venom toxicity and antivenom efficacy.

Since the phenol present in antivenoms is toxic to *A. salina*, we introduced a dialysis step to remove this preservative. Then, the antivenom was concentrated (in our case by freeze-drying followed by resuspension in water) to attain the same protein concentration as the original antivenom. We recommend using this, or a similar, protocol when testing phenol-containing antivenoms for their neutralizing efficacy using the *A. salina* model. This is not necessary when tests are done in mice because phenol, at the concentration used in antivenom, is not toxic to mice.

In conclusion, our observations strongly suggest that the *A. salina* model is a promising candidate for reducing reliance on mice in snake venom research and antivenom quality control. Future investigations should build upon these findings, addressing potential limitations and expanding the scope of *A. salina* in venom research and antivenom development.

## Ethical statement

This study was approved by the Institutional Committee for the Care and Use of Laboratory Animals (CICUA) of Universidad de Costa Rica (reference numbers 82-08 and 39-20).

## CRediT authorship contribution statement

**Xavier Araya:** Writing – review & editing, Writing – original draft, Methodology, Investigation, Conceptualization. **Mitchel Okumu:** Writing – review & editing, Writing – original draft, Methodology. **Gina Durán:** Writing – review & editing, Methodology, Investigation. **Aarón Gómez:** Writing – review & editing, Writing – original draft, Methodology, Investigation. **José María Gutiérrez:** Writing – review & editing, Writing – original draft, Project administration, Funding acquisition. **Guillermo León:** Writing – review & editing, Writing – original draft, Project administration, Methodology, Investigation, Funding acquisition, Conceptualization.

## Declaration of competing interest

The authors declare the following financial interests/personal relationships which may be considered as potential competing interests: Gina Durán, Aarón Gómez, José María Gutiérrez and Guillermo León work at Instituto Clodomiro Picado, University of Costa Rica, where the antivenom used in this study is manufactured.

## Data Availability

Data will be made available on request.
